# Supplementary figures and images for: Genome-Wide Scan of Wool Production Traits in Akkaraman Sheep
Source: Genes (Basel). 2023 Mar 14;14(3):713. doi: 10.3390/genes14030713 (PMC10048666; doi:10.3390/genes14030713)

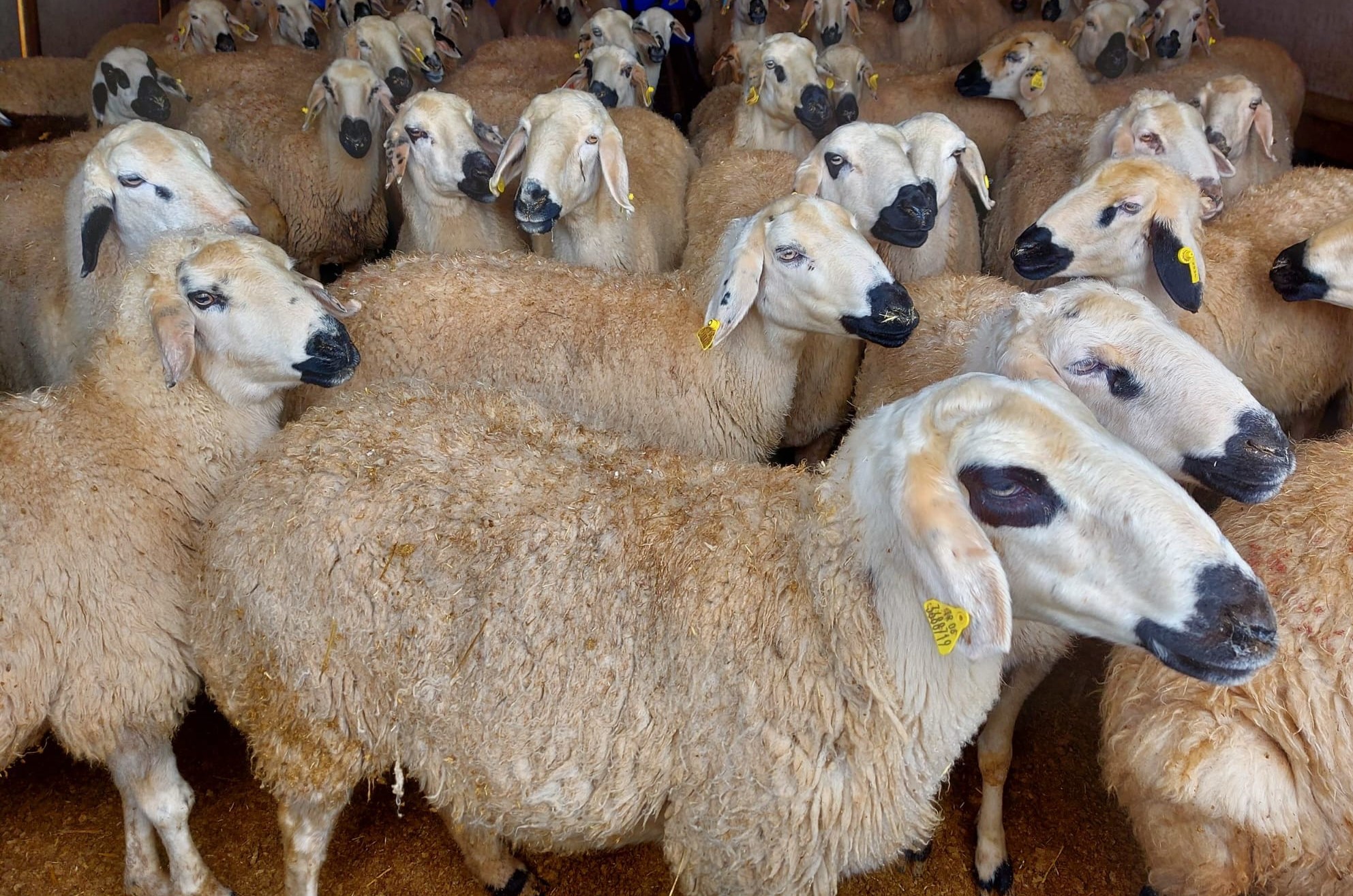

Supplement: Supplementary file 1 [file genes-14-00713-s001.zip › genes-2092781-supplementary/Supplementary Figure S1.jfif]

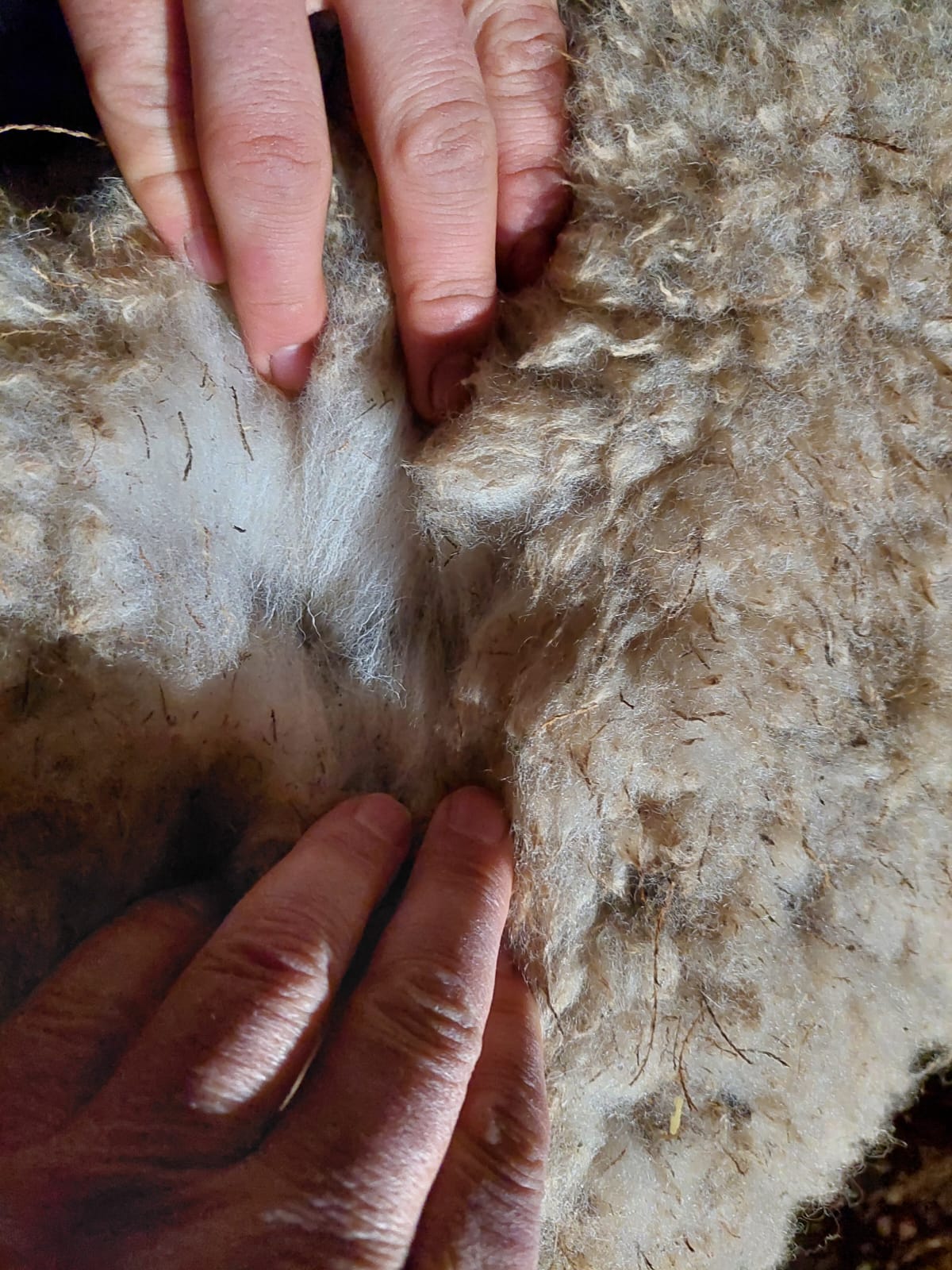

Supplement: Supplementary file 1 [file genes-14-00713-s001.zip › genes-2092781-supplementary/Supplementary Figure S2.jfif]
